# Do Health and Forensic DNA Databases Increase Racial Disparities?

**DOI:** 10.1371/journal.pmed.1001100

**Published:** 2011-10-04

**Authors:** Peter A. Chow-White, Troy Duster

**Affiliations:** 1 School of Communication, Simon Fraser University, Vancouver, British Columbia, Canada; 2 Department of Sociology, New York University, New York, New York, United States of America; 3 Department of Sociology, University of California, Berkeley, California, United States of America

## Abstract

Peter Chow-White and Troy Duster examine the question of whether the "digital divide" in health and forensic DNA databases is contributing to racial disparities.

**Summary Points**

- The issue of the digital divide is a growing concern in health and forensic DNA databases, reflecting structural disparities in biomedical research and policing.
- Over the last decade, the majority of DNA samples in population studies are from individuals of European origin. Individuals from Asian, African, Latino, and aboriginal groups are underrepresented.
- Forensic DNA databases are growing to mirror racial disparities in arrest practices and incarceration rates. Individuals from African American and Latino groups are overrepresented in forensic from health DNA databases.
- Currently, there is little recognition in national and international public policy circles about the “digital divide” in health and law enforcement databases.
- To avoid reproducing structural patterns of racial inequality, regulators, policy makers, scientists, and law enforcement officials need to address these disparities by supporting policies and mechanisms designed to better protect individuals and groups through institutional practices, law, and securely encrypted digital codes.

In the 1990s, scientists, social advocates, policy makers, and entrepreneurs debated the promises and perils of emerging digital technologies that could bring about enormous and wide-reaching changes in society. One set of debates revolved around the Internet while the other focused on genomics. Both framed the contours of the technological and social shifts in terms of the digital divide [1]. Politicians and entrepreneurs argued that connection to the Internet would be a basic necessity for all citizens and create a better society. Policy makers were concerned that women, racial and ethnic minorities, the working class, and unemployed citizens would be left out of the network revolution if they didn't connect to the Internet. Scientists such as Walter Gilbert worried that the increase of biological information in databases from new genomic technologies would divide the world into haves and have-nots [2]. Instead of new technologies ameliorating social inequalities, many feared they would exacerbate them. In both cases of technological innovation, “access” would be the key to creating a more equitable, just, and democratic society. However, as the decade has unfolded, it has now become increasingly evident that who is in DNA databases and who is using them and why requires as much attention as who is connected to them.

Since the completion of the Human Genome Project, there has been a global boom in DNA databases. Scientists, entrepreneurs, medical facilities, and law enforcement officials have uploaded a torrent of digital DNA information to public, commercial, university, medical, and law enforcement databases. Biomedical scientists extol the benefits of DNA for helping lead researchers to the genetic origins of complex diseases [3]. Law enforcement officials in Europe and North America claim that the expansion of DNA collection increases the ability to identify and apprehend suspects of crimes such as rape and murder [4]. Consumers pay biotechnology entrepreneurs to collect their DNA for the purpose of creating a personal medical profile and determining their genetic ancestry [5]. While the debates about the relationship between genome information and race rose and fell during the last decade, and the uses of DNA spread to different institutional contexts, there has been less attention paid to an emerging digital divide between health and forensic DNA databases. While we approach this intersecting set of issues from the perspective of North America, we hope that it has insights for other contexts.

## DNA Databases and Health

Discussions about access and ownership of genome information in the 1990s turned to debates about the biological versus social nature of race, the reification of race, the role of race in scientific research, and the validity of race as a biological variable in science [6]–[11]. Stakeholders paid less attention to the extent to which different social groups were being included in genome databases and in studies about the genetic origins of human disease. By the end of the first decade of the new century, the deluge of genome information into digital databases was dramatically uneven by class and race, creating a digital divide in genomic data.

Historically, scientists, advocates, and politicians have instituted progressive policy initiatives, such as US congressional legislation in 1993 and 2000, mandating researchers to include people from diverse racial and ethnic backgrounds in publically funded studies. This is an ironic state of affairs as there has also been a sharp increase in articles that study genetic differences between racial and ethnic groups as well as articles that report health disparities between them. Ioannidis and colleagues published data from a meta-study of genome-wide association studies research up to the mid 2000s that shows the vast majority of samples used in the studies are from European individuals [12]. The disparity in samples is echoed in biomedical and clinical research, as there is a dearth of epidemiological studies on nonwhite populations [13]–[15]. New research finds that over the last decade the majority of DNA samples in population studies are from individuals of European origin [16]. Individuals of Asian and African ancestries are underrepresented and there are very few DNA samples from Latino and aboriginal peoples used in the production of knowledge about genome variation, medical conditions, and human health. This disparity is also accumulating in the private databases of direct-to-consumer genomics companies. For example, the racial makeup of the 100,000+ samples [17] in the Google-Genentech direct-to-consumer genomics company, 23andMe, database is overwhelmingly white [18].

This form of digital information inequality has consequences for database representation and for the production of scientific and health knowledge. Despite the turn to difference in genomics, historical racial disparities in medical and scientific research appear to be reproducing in DNA databases as genome information tilts in the direction of white samples. This trend has generated a remarkable development in nations outside the orbit of Western science, with “national genomic sovereignty” emerging as a banner under which many countries are now pursuing research on “their own” people. Most notably, India, Mexico, and the pan-Asian Consortium are creating their own national genomic databases [19]. While these developments address the earlier imbalance created by European and North American domination of the DNA databases, they do nothing to deal with the sharp social and cultural digital divides between the health and forensic databases inside a country.

## DNA Databases and Forensics

Forensic DNA databases are growing to mirror racial disparities in arrest practices and incarceration rates. For example, over the last three decades, the population of American prisons has dramatically risen to comprise more than two million people. This increase has been accompanied by a dramatic shift in its racial composition as many African Americans and Latinos are incarcerated because they reside in communities where police systematically practice “buy and bust” operations. These types of police practices are rare in white communities where drug use is relatively higher than in African American and Latino neighborhoods [20]. Because of the differences in policing operations, the DNA databases held by law enforcement mirror the incarceration rates for African American and Latinos. This situation may be becoming worse as the collection of DNA creeps from convicted felons to individuals who are simply arrested. As more and more arrestees are locked into national DNA forensic databases, we will see an increasingly volatile intersection of race and ethnicity and “the CSI effect” (*CSI: Crime Scene Investigation* is a popular American crime drama television series where show creators often portray DNA as easily obtainable and rapidly sequenced by law enforcement and jurors treat DNA forensics as an irrefutable form of evidence) [21],[22].

Some advocates for DNA collection claim, falsely, there is no difference between DNA and “fingerprinting” and often use the misleading term “DNA fingerprint.” For example, in a story about familial DNA searching on the popular US primetime television news magazine show *ABC Nightline*, the former Attorney General for the state of California's Department of Justice argued that DNA is no different than a fingerprint in terms of its invasion of an individual's privacy [23]. The reporter interviewing the Attorney General failed to ask a critical but simple question: “If you say that DNA has no more invasion of privacy than a traditional fingerprint, tell us how a literal fingerprint would have lead to the suspect?” The right answer would be that DNA far exceeds the physical fingerprint as it contains much more information about potential or existing genetic diseases or genetic susceptibilities, and has been successfully used to capture kin relations through a technique called “familial searching.” It is not only a unique identifier; it is a network identifier.

In the digital age DNA is the biomedical equivalent of the social networking service Facebook. An individual is no longer the sum of a unique identifier, such as an actual fingerprint. Her identity is genetically related to other people's identities that are related to that individual. Also, once DNA and personal information enter the database surveillance net, an individual loses control over her genomic and individual identity. It is then subject to data mining of scientists, entrepreneurs, marketers, “friends,” and Google. DNA holds information such as disease risk of an individual and their past, present, and future family relationships. There may be much more we do not know about as genome research continues into the putative links between DNA and behavior. The last two decades have witnessed a sharp increase in studies that claim DNA markers as indicators of intelligence and violence, and even political orientation [24]. The networks of relationships between genes and the environment are so complex that the genome may hold more personal and family information than we are presently aware.

## Conclusion

Stakeholders in different domains such as health and law enforcement increasingly produce information from statistical techniques and data mining of DNA databases. There are enormous social benefits and risks associated with new DNA technologies. The pressure to employ these new technologies comes from the desire to improve health knowledge and protection of citizens. However, they also identify, sort, and compare social groups in terms of expected value or risk [25]. We should be especially concerned about the disparities in DNA databases while they are expanding and the technology is diffusing at a rapid rate. The British Nuffield Council on Bioethics recently released a report on personalized medicine [26]. While the authors addressed a number of risks in personal genetic profiling, the report was silent on the nature of the DNA information in DNA databases. At the global level, there are efforts to network nationally based forensic DNA databases. Advocacy groups suggest the variation between the different DNA information systems raises concerns for privacy and civil rights [27]. The recent National Academy of Sciences/National Research Council (NAS) [28] and Nuffield reports are steps in the right direction. The NAS report found that the system of forensic science and labs need significant improvement to resolve widespread discrepancies between local labs in terms of program underfunding and lack of standardization in certifying staff. Additionally, the report found that DNA match technology is largely uncertain in its reliability and validity, and the effects of human bias and error need to be determined. Still, the 2009 NAS report gave a complete pass to DNA as the “gold standard” for forensic identification and profiling without offering a critique of its uses and abuses. A Nuffield report that addressed forensic science [29] concluded that the over-representation of minority groups is related to policing practices. In this sense, the work that goes on in the lab is a secondary concern to the policing and collection practices that disproportionally gather DNA from nonwhite, poor, and working class populations in the digital surveillance net. The European Union has generally blocked and even overturned moves to extend and retain DNA—and has further ruled that sample data must be destroyed if the person was not convicted [30],[31].

Currently, there is little discussion in national and international public policy circles about the racial digital divide between health and law enforcement databases. A recent article in *The Washington Post* about debates regarding DNA collection is a good example [32]. There is no mention in the article, or in the debate that apparently preceded it (note the lopsided votes), of a digital divide. The first and foremost step in addressing the problem is recognizing that this is an issue. That is, we cannot address the problem unless or until there is awareness. What is needed now in national contexts such as the US and the UK and at the international level are new reports that deal with the disproportionate databasing of DNA within and across institutional contexts.

These types of digital divides risk exacerbating legacies of inequality in biomedical research and policing. They are cases of digital technology intensifying old divides and creating new ones in ways that have not been fully appreciated. It is a reminder that while the technology itself may not be “good” or “bad,” in practice, it is rarely if ever neutral. It is up to us to decide how we go about using and innovating new DNA technologies. To avoid reproducing structural patterns of racial inequality, regulators, policy makers, and users (such as scientists and law enforcement officials) need to address these disparities by supporting policies and mechanisms designed to better protect individuals and groups through institutional practices, law, and securely encrypted digital codes. Ultimately, the over-arching concerns that should guide these developments revolve around how these databases are used. Up until now, this use has led too many to ignore the equally important digital divide between those in health and forensic gene pools.
